# Bibliometric analysis of post-traumatic stress disorder in forensic medicine: Research trends, hot spots, and prospects

**DOI:** 10.3389/fpsyg.2022.1074999

**Published:** 2023-01-16

**Authors:** Weihao Zhu, Yingmin Li, Xiaoying Ma, Huihuang Yang, Zhen Wang, Rui Shi, Weibo Shi, Bin Cong

**Affiliations:** Hebei Key Laboratory of Forensic Medicine, Collaborative Innovation Center of Forensic Medical Molecular Identification, College of Forensic Medicine, Hebei Medical University, Shijiazhuang, China

**Keywords:** forensic medicine, post-traumatic stress disorder, stress response area, biomarker, COVID-19

## Abstract

**Background:**

Post-traumatic stress disorder (PTSD) has various risk factors, complex pathogenesis, and diverse symptoms, and is often comorbid with other injuries and diseases, making forensic diagnosis difficult.

**Methods:**

To explore the current research status and trends of PTSD, we used the Web of Science Core Collection databases to screen PTSD-related literature published between 2010 and 2021 and CiteSpace to perform bibliometric analysis.

**Results:**

In recent years, PTSD-related research has grown steadily. The countries and institutions with the most research results were the United States and England, and King’s College London and Boston University, respectively. Publications were identified from 2,821 different journals, including 13 forensic-related journals, but the journal distribution was relatively scattered and there was a lack of professional core journals. Keyword co-occurrence and clustering identified many hot topics; “rat model,” “mental health,” and “satisfaction” were the topics most likely to have a clear effect on future research. Analysis extracted nine turning points from the literature that suggested that neural network centers, the hypothalamic–pituitary–adrenal axis, and biomarkers were new research directions. It was found that COVID-19 can cause severe psychological stress and induce PTSD, but the relationship needs further study. The literature on stress response areas and biomarkers has gradually increased over time, but specific systemic neural brain circuits and biomarkers remain to be determined.

**Conclusion:**

There is a need to expand the collection of different types of biological tissue samples from patients with different backgrounds, screen PTSD biomarkers and molecular targets using multi-omics and molecular biology techniques, and establish PTSD-related molecular networks. This may promote a systematic understanding of the abnormal activation of neural circuits in patients with PTSD and help to establish a personalized, accurate, and objective forensic diagnostic standard.

## Introduction

There is a current increase in wars and local social unrest, terrorist attacks, violent incidents, major traffic accidents, natural disasters, and personal injury events that cause substantial stress and suffering (Groll et al., 2020). People who have experienced major threatening and catastrophic events may experience the delayed emergence and persistence of post-traumatic stress disorder (PTSD), a mental disorder characterized by abnormal mental behavior and psychological disorders (Boyd et al., 2018; Ben et al., 2019). PTSD has a high prevalence and is associated with a high suicide risk; its main clinical manifestations are re-experiencing traumatic events, persistently increased alertness, and emotional numbness and avoidance behavior (Yehuda et al., 2015). Epidemiological surveys show that the incidence of PTSD is 5–10%; however, the risk is higher in some groups, such as military personnel and rape survivors (≥25–50%), and the incidence is twice as high in women than in men (Yehuda et al., 2015).

PTSD prevalence varies markedly across populations and countries due to cultural differences, geographically specific distribution of trauma type and severity (Yehuda et al., 2015). Clinical research found that there are many risk factors related to PTSD, including the victim’s family history and family environment, gender, age, living habits, personality characteristics, heredity, history of psychological and physical trauma, education level, the nature of the traumatic event and post-traumatic interventions, etc. (Boyd et al., 2018). When an individual experiences trauma, a systemic stress response may occur, resulting in severe secondary central nervous system damage (Herman, 2012), cardiovascular system damage (Lampert, 2016), endocrine and metabolic disorders (Lamounier-Zepter et al., 2006), digestive system damage (Ogasawara et al., 2020), and even death. In forensic medicine, stressors such as social and psychological factors can also damage the body *via* stress mechanisms (Zhang Y. et al., 2014; Lampert, 2016; Romero-Ramirez et al., 2017), such as the stress response after mechanical injury (Tan et al., 2018; Yi et al., 2019). This mechanism may have two aspects. First, the mechanical injury itself causes an excessive homeostatic load through the sympathetic adrenal medulla system and the hypothalamic–pituitary–adrenal (HPA) cortical system (Rubin et al., 2016; Albrechet-Souza and Gilpin, 2019; Kim et al., 2019). Second, the injury may cause an adverse psychological reaction, and psychological stress may excessively challenge homeostasis (Gallagher and Barbe, 2022). When the stress response reaches a specific level, it can cause tissue cell damage. Therefore, it is important to increase the awareness of PTSD among forensic practitioners and to increase research on PTSD.

In recent years, there has been an increase in studies on PTSD; however, these studies lack a comprehensive perspective. Bibliometric analysis could help to resolve this problem. Bibliometrics is an interdisciplinary science that quantitatively analyzes all knowledge carriers using mathematical and statistical methods. Through statistical analysis of published literature, it is possible to determine citation relationships between different publications in a particular field, the current state of research, and research trends, and thus to evaluate the contributions of different countries and researchers (Li et al., 2022). The present study aimed to present a comprehensive overview of research trends and hot spots in the field of PTSD from 2010 to 2021 using bibliometric analysis, and to provide directions for future research.

## Materials and methods

### Database and search strategy

In this retrospective study, we searched the Web of Science Core Collection databases (including SCI-EXPANDED, SSCI, A&HCI, CPCI-S, CPCI-SSH, ESCI, CCR-EXPANDED, IC^1^) for relevant literature on May 1, 2022. To take into account the recall rate and precision rate of retrieving PTSD-related literature, we did not expand the proprietary subject headings. The search strategy was as follows: TS = (post-traumatic stress disorder OR PTSD AND (forensic OR legal medicine)). Publications were retrieved for the period January 1, 2010, to December 31, 2021.

### Inclusion and exclusion criteria

**Figure 1:**
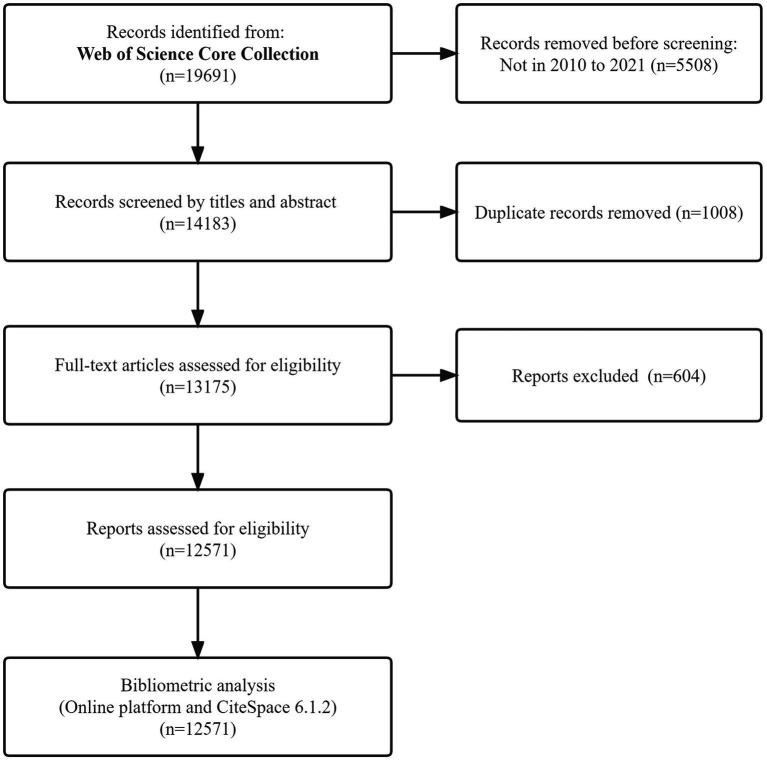
The search process.

We included in the analysis PTSD risk factors, etiology, injury mechanisms, preventive diagnosis, treatment methods, and other factors. All types of publications in the Web of Science Core Collection databases were included. Non-research articles on humans, publications that did not mention PTSD, and those on related diseases were excluded. Articles were evaluated by three independent investigators who considered the title, author, abstract, keywords, publication year, and journal. In the event of any disagreement, the majority view was accepted in deciding whether to include a study. There were no restrictions on the language of publications. Figure 1 illustrates the search process. In this flowchart, we conducted a literature search and screening according to the Preferred Reporting Items for Systematic Reviews and Meta-Analyses (PRISMA) guidelines (Page et al., 2021).

### Data analysis

Bibliometric analysis was conducted by exporting and importing eligible records using CiteSpace (version 6.1.2^2^) following record screening by the researcher. To analyze the status and trends of PTSD publications worldwide, we selected the following indicators: published articles per year, country co-occurrences, institution co-occurrences, top 10 cited journals (Web of Science provides journal impact factors and Journal Citation Reports (JCR) partitions), top 10 leading authors and prolific authors, top 10 co-cited literatures, keyword co-occurrence, and burst words.

We created a country co-occurrence map, institution collaboration map, statistics table of top 10 journals, author collaboration co-occurrence map and top 10 author statistics table, author co-citation map, literature co-citation map and turning point articles statistics table, keyword frequency statistics and keyword clustering map, and a top 25 burst words map. These demonstrated the cross-references among journals, collaboration among countries, keyword co-occurrences, new research hotspots, and the contributions of researchers worldwide.

We selected three subtopics (stress response areas (SRA), biomarkers, and COVID-19) as hotspots for secondary analysis based on the publication volume and growth trend of PTSD-related literature. We manually screened each document and classified it into SRA, biomarkers, COVID-19, and other categories. The selection criteria for SRAs were amygdala OR hypothalamus OR prefrontal cortex OR hippocampus OR reticular formation OR cingulate gyrus OR septal area OR hypothalamic–pituitary–adrenal axis OR HPA OR HPA axis; the selection criteria for biomarkers were molecular indicators OR genes OR proteins OR cytokines; the screening criteria for COVID-19 were COVID-19 OR coronavirus disease 2019 OR novel coronavirus pneumonia OR NCP. Documents that did not meet these three screening criteria were classified into the “other” category. Then, the grouped publications were entered into CiteSpace and the relevant indicators were statistically analyzed. Simultaneously, we manually screened and calculated the publication volume and proportion of each SRA-related publication in the SRA group for different periods.

## Results

Implementing the strategy described above, 19,691 publications were retrieved and individually evaluated. We removed 5,508 papers that were not in 2010–2021, 1,008 duplicates by reading titles and abstracts and 604 papers that were not relevant after full-text reading by reviewers. Finally, 12,571 publications were selected for bibliometric analysis. Most of these were articles (*n* = 10,248, 81.52%), followed by literature reviews (*n* = 1804, 14.35%), and editorial materials, letters, and papers (*n* = 519, 4.13%).

### Number and growth trend of annual publications

**Figure 2:**
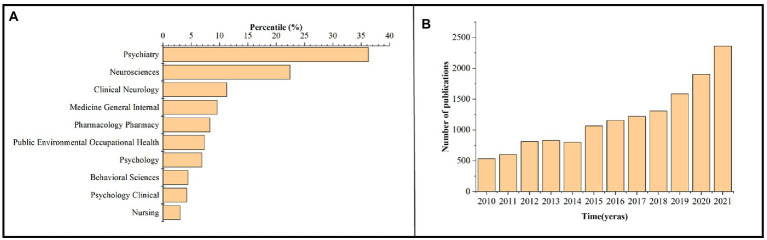
Publications on PTSD from 2010 to 2021. **(A)** Subject categories distribution. **(B)** Annual publications quantitative distribution.

We retrieved and screened 12,571 PTSD-related documents published between 2010 and 2021 in the field of forensic medicine from the Web of Science Core Collection. The top subject categories were psychiatry (36.277%) and neurosciences (22.422%) (Figure 2A). The volume of publications gradually increased each year except for 2014, when the volume of publications declined slightly compared with the previous year. Since 2018, the number of publications has substantially increased (Figure 2B).

### Primary countries or regions

**Figure 3:**
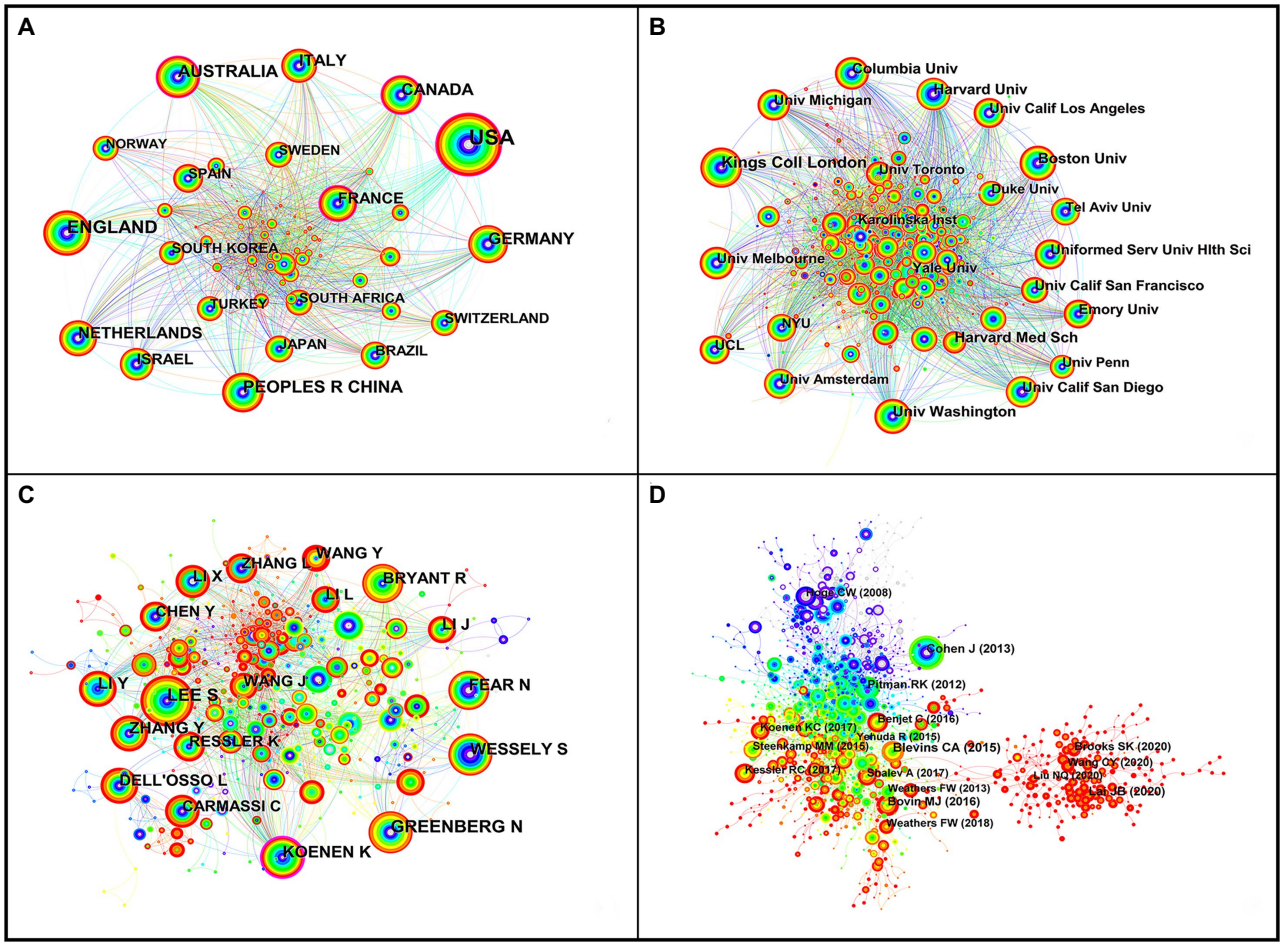
The bibliometric analysis. **(A)** primary countries or regions; **(B)** primary institutions; **(C)** leading authors and prolific authors; **(D)** literature co-citation. The nodes size represents the quantity of papers **(A–C)** or co-citation **(D)**; the purple outer circle nodes indicate that the number of studies in countries or regions **(A)** or institutions **(B)** or authors **(C)** or co-cited literature **(C)** may continue to increase, and nodes with orange or gray outer circles indicate that they has received attention in the past, but is no longer hot spots; the thickness of the line between the nodes represents degree of connection **(A–C)** or co-citation **(D)**; the warmer the color of the line, the closer the interconnection time.

A total of 144 countries or regions participated in PTSD-related research (Figure 3A); those with more published literature had higher centrality. Of the 12,571 publications, researchers in the United States published the most, with 5,273 publications, ranking first; researchers in England ranked second with 1,297 publications, followed by Australia (866), the People’s Republic of China (816), Germany (735), Canada (720), Italy (568), the Netherlands (546), France (502), and Israel (380). Figure 3A shows that the top 10 countries or regions had the most mutual lines; in other words, these articles were more interconnected (greater cross-references).

### Primary institutions

A total of 578 institutions participated in research on PTSD. Researchers from King’s College London published the most publications, with 378, ranking first; researchers at Boston University ranked second, with 240 publications, followed by Harvard Medical School (228), Columbia University (202), University of Washington (199), Harvard University (192), University of California San Diego (186), University of Melbourne (184), Emory University (172), and Yale University (171) (Figure 3B). Publications from the top 10 institutions accounted for 17.12% (2,152/12,571) of the total publications.

### Primary journals

The 12,571 PTSD-related publications were published in 2821 journals, of which 280 published more than 10 publications and 29 published more than 50. The top 10 journals in terms of publication volume accounted for 14.01% (1761/12,571) of the total publication volume. All publications were in English. The 5-year impact factor range was 1.470–8.396 and the JCR partition range was Q1–Q3 (Table 1). Of the 2,821 journals, 13 were related to forensic medicine, namely the Journal of Forensic Psychiatry and Psychology, Journal of Forensic Science, International Journal of Forensic Mental Health, Journal of Forensic and Legal Medicine, Journal of Forensic Psychology Practice, Journal of Forensic Nursing, Journal of Forensic Practice, Australian Journal of Forensic Science, Forensic Science International, Journal of Forensic Psychology Research and Practice, International Journal of Legal Medicine, Journal of Legal Medicine, and Romanian Journal of Legal Medicine. The number of these journals published accounted for 0.45% (56/12,571) of the total number of publications.

**Table 1:** Top 10 journals contributed to scientific research on PTSD.

Journal	Region	Language	5 year IF	JCR partition	Articles	Percentage of total
Military Medicine	United States	English	1.470	Q3	273	2.173
Plos One	United States	English	3.788	Q2	204	1.626
European Journal of Psychotraumatology	England	English	5.577	Q2	192	1.529
Frontiers in Psychiatry	Switzerland	English	4.409	Q2	185	1.469
Journal of Affective Disorders	Netherlands	English	5.515	Q1/Q2	185	1.469
Psychological Medicine	United States	English	8.396	Q1	161	1.281
Psychiatry Research	Netherlands	English	3.405	Q2/Q3	158	1.259
Frontiers in Psychology	Switzerland	English	3.618	Q2	148	1.177
International Journal of Environmental Research and Public Health	Switzerland	English	3.789	Q1/Q2	128	1.019
BMC Psychiatry	England	English	4.514	Q2	127	1.012

### Leading authors and prolific authors

The 12,571 PTSD-related publications featured 671 authors, of which 144 authors published ≥10 papers, 40 authors published ≥30 papers, and 12 authors published ≥50 papers. Most articles were published by S. Lee, accounting for 0.71% (89/12,571) of the total published articles (Table 2). As shown in Figure 3C, the studies published by N. Greenberg, N. Fear, and S. Wesley, co-occurred the closest. S. Lee, Y. Li, L. Dell’osso, Y. and Zhang shared the most co-occurrences (only the top 10 authors were compared). Cross-citations between authors were calculated using CiteSpace and the results are shown in Table 3. R.C. Kessle, ranked first, with 2,381 citations; E.B. Foa ranked second, with 1,485 citations; F.W. Weathers ranked third, with 1,465 citations.

**Table 2:** Top 10 prolific authors on PTSD.

Author	Publications	Centrality	Year	Region	Institution
Lee S	89	0.07	2011	South Korea	1. Ewha Womans Univ, Ewha Brain Inst 2. Ewha Womans Univ, Dept Brain & Cognit Sci
Greenberg N	79	0.02	2010	England	1. Kings Coll London, Kings Ctr Mil Hlth Res 2. Kings Coll London, Acad Dept Mil Mental Hlth
Fear N	67	0.02	2010	England	1. Kings Coll London, Kings Ctr Mil Hlth Res 2. Kings Coll London, Acad Dept Mil Mental Hlth
Wessely S	65	0.02	2010	England	1. Kings Coll London, Kings Ctr Mil Hlth Res 2. Kings Coll London, Acad Dept Mil Mental Hlth
Koenen K	64	0.21	2011	United States	Columbia Univ, Mailman Sch Publ Hlth, Dept Epidemiol
Li Y	61	0.05	2012	Peoples R China	Chinese Acad Sci, Inst Psychol, Key Lab Mental Hlth
Zhang Y	59	0.03	2010	United States	1. DVA Med Ctr, Ctr Imaging Neurodegenerat Dis 2. Univ Calif San Francisco, Dept Radiol
Bryant R	58	0.09	2010	Australia	Univ New South Wales, Sch Psychol
Dell'osso L	56	0.04	2011	Italy	Univ Pisa, Dept Clin & Expt Med
Li X	52	0.01	2010	Australia	Univ Queensland, Queensland Brain Inst

“Year” indicates the year in which the author first published PTSD-related articles; The numbers in “Institution” indicate different institutions of the author.

**Table 3:** Top 10 highly co-cited authors on PTSD

Author	Co-citations	Centrality	Region	Institution
Kessler RC	2381	0.01	United States	Harvard Med Sch, Dept Hlth Care Policy
Foa EB	1485	0.01	United States	Univ Penn, Sch Med, Dept Psychiat
Weathers FW	1465	0	United States	Boston Univ, Sch Med
Brewin CR	1175	0.01	England	1 UCL, Clin Psychol 2 Camden & Islington NHS Fdn Trust, Traumat Stress Clin
Breslau N	1127	0.01	United States	Michigan State Univ, Coll Human Med, Dept Epidemiol & Biostat
Yehuda R	1110	0.02	United States	1 Icahn Sch Med Mt Sinai 2 James J Peters VA Med Ctr
Hoge CW	941	0.03	United States	Walter Reed Army Inst Res
Bryant RA	932	0.03	Australia	Univ New South Wales, Sch Psychol
Beck AT	843	0	United States	Univ Penn, Aaron T Beck Psychopathol Res Ctr, Perelman Sch Med
Ehlers A	753	0.04	England	Univ Oxford, Dept Expt Psychol

“0” in the table means that when two decimal places are reserved, it is approximately equal to 0.

### Literature co-citation

Co-citation analysis of PTSD-related literature showed that 1,498 studies were co-cited, of which 612 articles were cited ≥10 times, 136 were cited ≥30 times, 48 were cited ≥50 times, and 6 were cited ≥100 times (Figure 3D). After calculating the centrality of the co-cited publications, we screened out those publications whose centrality was ≥0.05 and were co-cited ≥20 times as the turning point (Table 4).

**Table 4:** Turning point articles in the field of PTSD.

First author (year of publication)	Title	Co-citations	Total citations	Centrality	Journal
Bryant RA, 2017	Acute and Chronic Posttraumatic Stress Symptoms in the Emergendce of Posttraumatic Stress Disorder A Network Analysis	21	139	0.31	JAMA PSYCHIATRY
Brewin CR, 2014	Episodic Memory, Perceptual Memory, and Their Interaction: Foundations for a Theory of Posttraumatic Stress Disorder	24	211	0.2	PSYCHOLOGICAL BULLETIN
van Zuidenm, 2012	Glucocorticoid Receptor Pathway Components Predict Posttraumatic Stress Disorder Symptom Development: A Prospective Study	26	138	0.09	BIOLOGICAL PSYCHIATRY
Daskalakis NP, 2013	Endocrine Aspects of Post-traumatic Stress Disorder and Implications for Diagnosis and Treatment	22	123	0.08	ENDOCRINOLOGY AND METABOLISM CLINICS OF NORTH AMERICA
Ressler KJ, 2011	Post-traumatic stress disorder is associated with PACAP and the PAC1 receptor	57	523	0.07	NATURE
Brewin CR, 2010	Intrusive Images in Psychological Disorders: Characteristics, Neural Mechanisms, and Treatment Implications	34	617	0.07	PSYCHOLOGICAL REVIEW
Pitman RK, 2012	Biological studies of post-traumatic stress disorder	120	833	0.06	NATURE REVIEWS NEUROSCIENCE
Milad MR, 2009	Neurobiological Basis of Failure to Recall Extinction Memory in Posttraumatic Stress Disorder	54	860	0.06	BIOLOGICAL PSYCHIATRY
Shin LM, 2010	The Neurocircuitry of Fear, Stress, and Anxiety Disorders	53	1163	0.05	NEUROPSYCHOPHARMACOLOGY

Turning Point means articles with high centrality and high Co-citations (centrality ≥ 0.05 and co-citations ≥ 20).

### Top keyword co-occurrence

**Figure 4:**
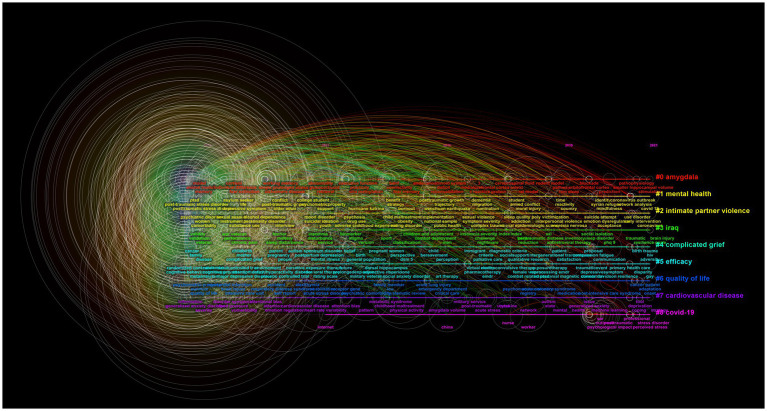
Keyword cluster analysis time diagram. Lines of different colors represent different clusters of keywords, and the circles size represents the number of articles where the keyword appears.

We used CiteSpace to analyze keyword co-occurrence, with keyword as the node type. We obtained 913 nodes, 4,546 links, and the density was 0.0109. The term “posttraumatic stress disorder (post-traumatic stress disorder, PTSD)” appeared most frequently, with 7,991 (3,658 + 2,354 + 1979) occurrences, ranking first; closely followed by the term “symptom,” which appeared 1776 times (Table 5). As can be seen from Table 5, 12 keywords were causes or symptoms of PTSD; three words were related to the participants of PTSD studies, namely veterans, survivors, and children. To identify the most popular PTSD research topics, we conducted a keyword clustering analysis. The highest clustering strength (the largest cluster) was “amygdala,” followed by “mental health,” “intimate partner violence,” and “Iraq” (Figure 4).

**Table 5:** Top 20 keywords on PTSD.

Keywords	Articles	Centrality	Year
Posttraumatic stress disorder	3658	0	2010
Post-traumatic stress disorder	2354	0	2010
PTSD	1979	0	2010
Symptom	1776	0.01	2010
Depression	1745	0	2010
Mental health	1681	0.01	2010
Prevalence	1408	0.01	2010
Disorder	1143	0.01	2010
TRAUMA	1058	0	2010
Risk factor	852	0	2010
Anxiety	775	0	2010
Impact	774	0	2010
Risk	699	0.01	2010
Health	675	0	2010
Quality of life	670	0.01	2010
Veteran	569	0.01	2010
Exposure	560	0.01	2010
Survivor	535	0	2010
Metaanalysis	503	0.01	2010
Children	487	0.01	2010

“0” in the table means that when two decimal places are reserved, it is approximately equal to 0; “Year” indicates the year in which the author first published PTSD-related articles.

### Burst words

**Figure 5:**
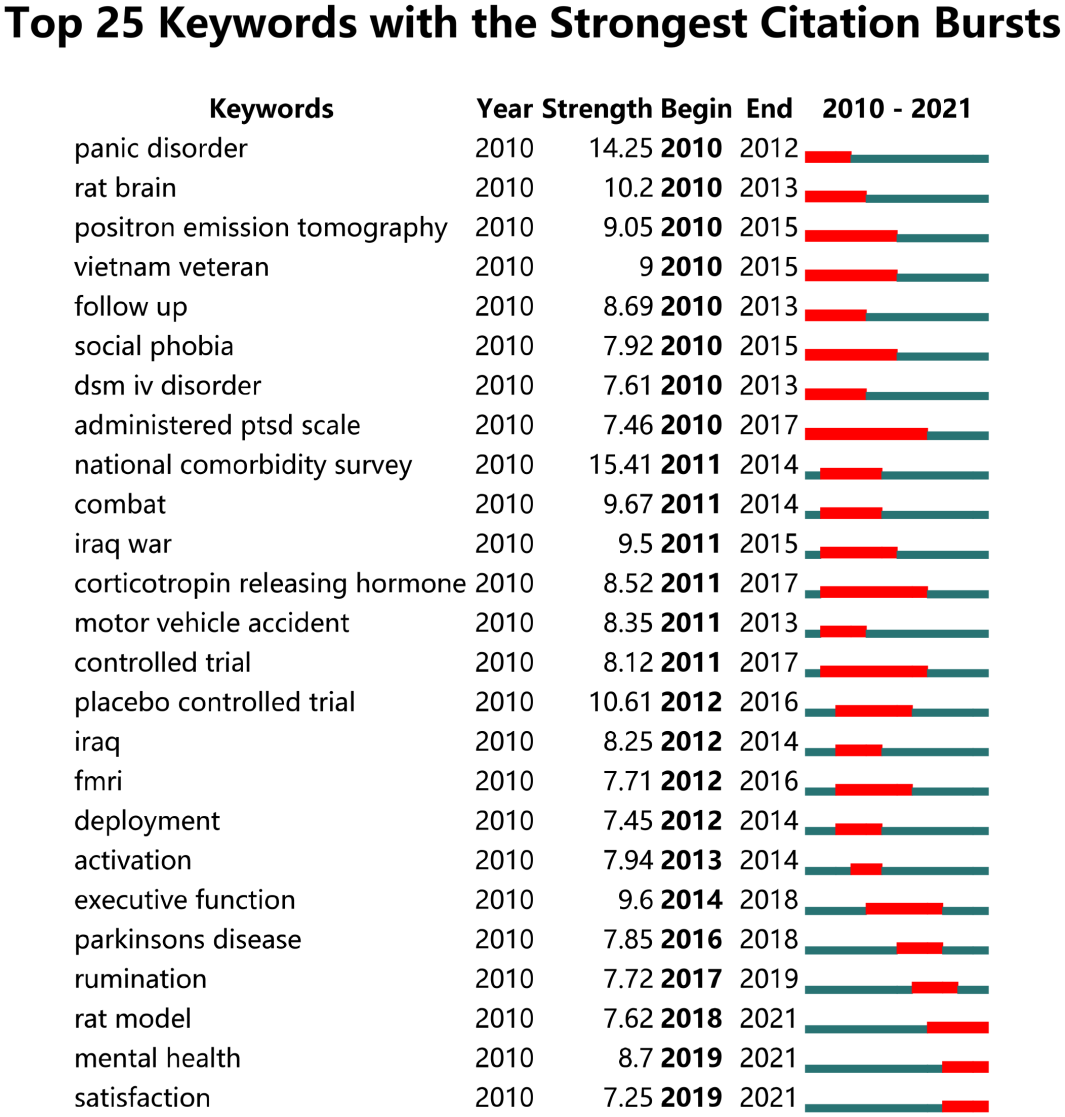
The top 25 keywords with the strongest citation bursts.

Analysis of the keywords extracted from PTSD-related literature produced 273 burst words, which were defined as keyword variables with large changes during this period. We extracted the top 25 keywords with the strongest citation bursts (Figure 5) and found that “rat model,” “mental health,” and “satisfaction” were the topics most likely to influence future research on PTSD in forensic medicine. “National comorbidity survey” was the strongest word, appearing in 2011–2014, with a burst intensity of 15.41; followed by “panic disorder,” with a burst intensity of 14.25; and “placebo-controlled trial,” with an intensity of 10.61.

### Annual number of SRA-and biomarker-related publications

**Figure 6:**
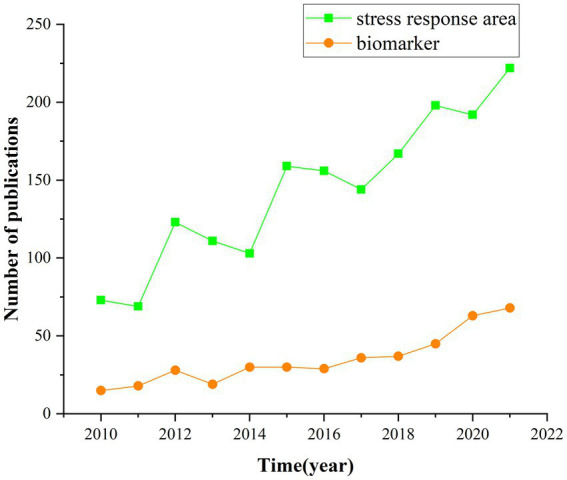
Annual articles number of SRA and biomarker.

After a further screening of PTSD-related literature, 1717 SRA-related articles and 418 biomarker-related articles were identified. From 2010 to 2021, the number of publications on SRAs and biomarkers generally showed an upward trend, except for a slight decline in the number of papers published in some years compared with the previous year. There were more SRA-related publications per year than biomarker-related publications (Figure 6). This indicates that researchers continue to explore the injury response site of PTSD, diagnostic indicators, and mechanisms of action.

### SRAs

The 1717 SRA-related publications were split into two groups (2010–2015 and 2016–2021) according to the period; 639 publications were in 2010–2015 and 1,078 in 2016–2021. We separately counted the number and proportion of publications related to different SRAs in the two time periods and compared the changes in related SRAs. Most publications were on the amygdala and prefrontal cortex, accounting for 48.46% (832/1717) and 48.17% (827/1717) of the total number of SRA-related publications, respectively. Table 6 shows that there were 2,772 publications related to different SRAs, which was much larger than 1717, suggesting that some publications reported studies of multiple SRAs. The SRAs with negative growth were reticular formation and septal area, and they appeared 2 and 1, respectively in the 2010–2015 period, indicating that stress may not have an obvious negative effect on these two SRAs, and that research interest in them is not high. In addition, except for cingulate gyrus, which had a growth rate of 16.67% (35–30)/30), the growth rates of other SRAs all exceeded 50% (Table 6).

**Table 6:** Literature analysis of different SPAs in 2010-2015 and 2016-2021.

SRAs	2010–2015 (*n*=639)		2016–2021 (*n*=1078)		Growth rate	Total (*n*=1717)	
	Number	Proportion	Number	Proportion		Number	Proportion
Amygdala	323	50.55%	509	47.22%	57.59%	832	48.46%
Hypothalamus	44	6.89%	70	6.49%	59.09%	114	6.64%
Prefrontal cortex	309	48.36%	518	48.05%	67.64%	827	48.17%
Hippocampus	235	36.78%	412	38.22%	75.32%	647	37.68%
Reticular formation	2	0.31%	0	0.00%	-200%	2	0.12%
Cingulate gyrus	30	4.69%	35	3.25%	16.67%	65	3.79%
Septal area	1	0.16%	0	0.00%	–100%	1	0.06%
Hypothalamic-pituitary-adrenal axis, HPA, HTPA axis	113	17.68%	171	15.86%	51.33%	284	16.54%

**Figure 7:**
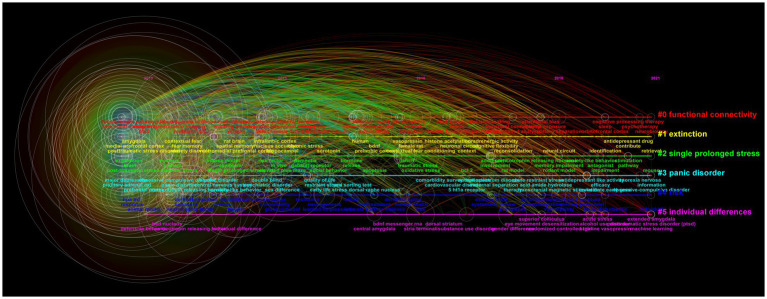
Time diagram of keyword clustering analysis of SRA.

**Figure 8:**
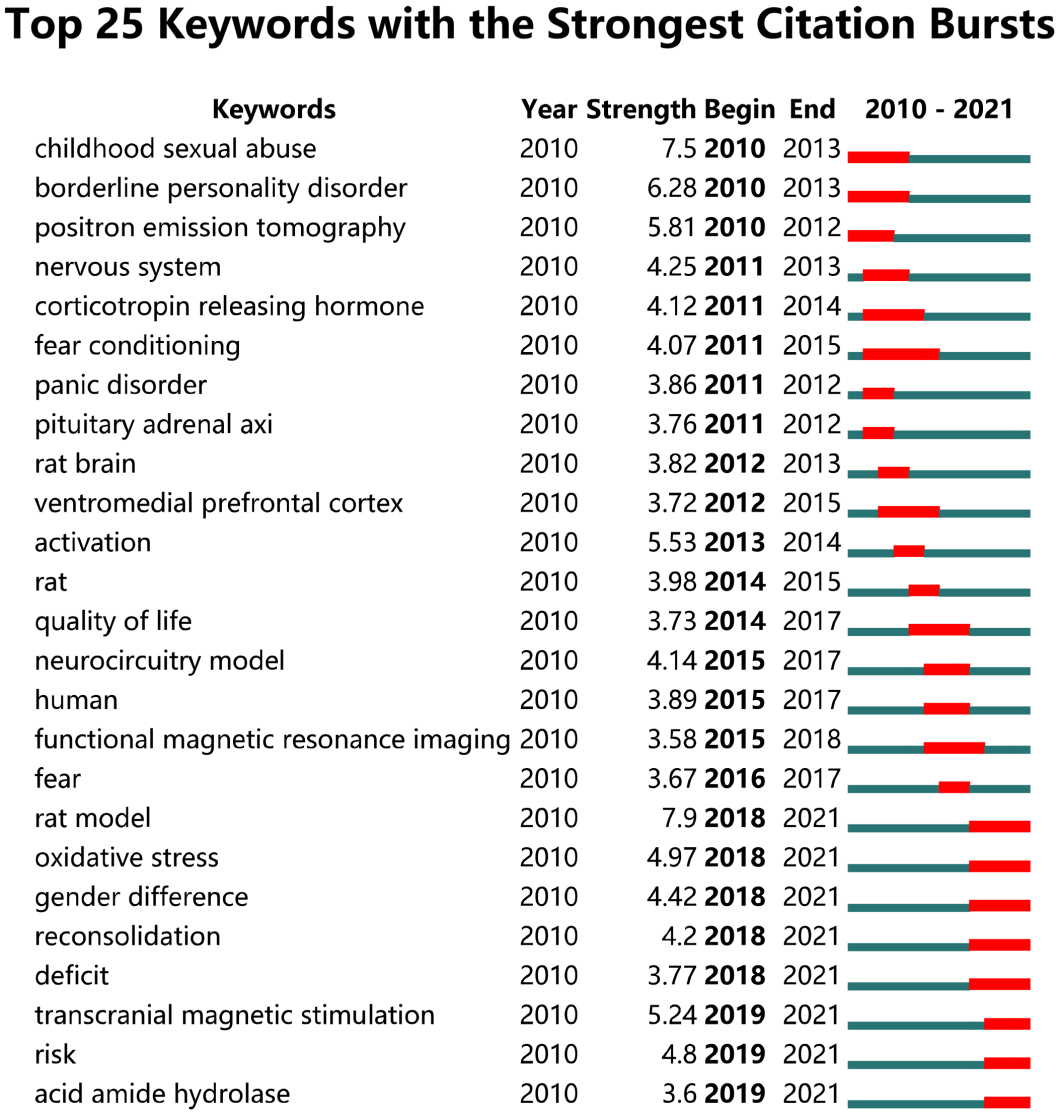
The top 25 keywords with the strongest citation bursts in SRA-related literature.

We used CiteSpace to analyze 1717 SRA-related publications, and screened out 504 keywords: 178 keywords with publication volume ≥ 10, 19 keywords with publication volume ≥ 100, and 6 keywords with publication volume ≥ 200, namely “posttraumatic stress disorder,” “post-traumatic stress disorder,” “prefrontal cortex,” “medial prefrontal cortex,” “amygdala,” and “PTSD.” The cluster analysis of keywords identified six clusters, namely “functional connectivity,” “extinction,” “single prolonged stress,” “panic disorder,” “risk,” and “individual differences” (Figure 7). Keyword extraction and analysis of SRA-related literature was performed, screening out the 70 strongest citation burst words. Analysis of the top 25 burst words (Figure 8) indicated that “rat model,” “oxidative stress,” “gender difference,” “reconsolidation,” “deficit,” “transcranial magnetic stimulation,” “risk,” and “acid amide hydrolase” were the topics most likely to influence future research on PTSD in forensic medicine. The strongest word was “rat model,” which appeared in 2018–2021, with a burst intensity of 7.9; followed by “childhood sexual abuse,” with a burst intensity of 7.5; and “borderline personality disorder,” with an intensity of 6.28.

### Biomarkers

We used CiteSpace to analyze the keywords from 418 biomarker-related publications and found 347 keywords. After excluding three keywords (“posttraumatic stress disorder,” “post-traumatic stress disorder,” “PTSD”), the 10 keywords with the highest frequency were extracted (Table 7). We counted the number of keywords that appeared in 2010–2015 and 2016–2021, and calculated changes in the top 10 keywords. The number of published articles for the top 10 keywords all increased to varying degrees; the publication volume for association had the largest growth rate, at 300%, and glucocorticoid receptor had the smallest growth rate, at 15.38% (Table 7).

**Table 7:** Top 10 key words of molecular markers in 2010–2014 and 2016–2021.

Key words	2010–2015	2016–2021	Growth rate	Total
Expression	22	45	104.55%	67
Depression	13	40	207.69%	53
Gene expression	11	30	172.73%	41
Animal model	15	25	66.67%	40
Anxiety	10	26	160.00%	36
DNA methylation	8	22	175.00%	30
Brain	10	20	100.00%	30
Association	6	24	300.00%	30
Glucocorticoid receptor	13	15	15.38%	28
Memory	8	20	150.00%	28

**Figure 9:**
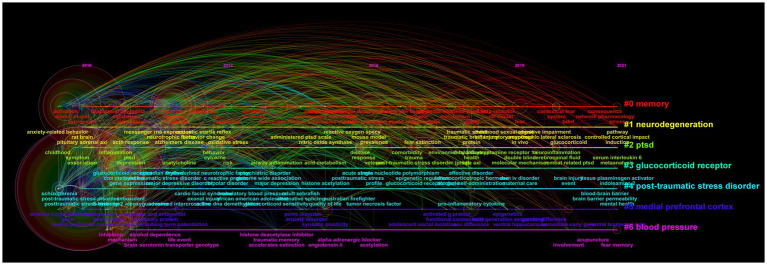
Time diagram of keyword clustering analysis of biomarker.

**Figure 10:**
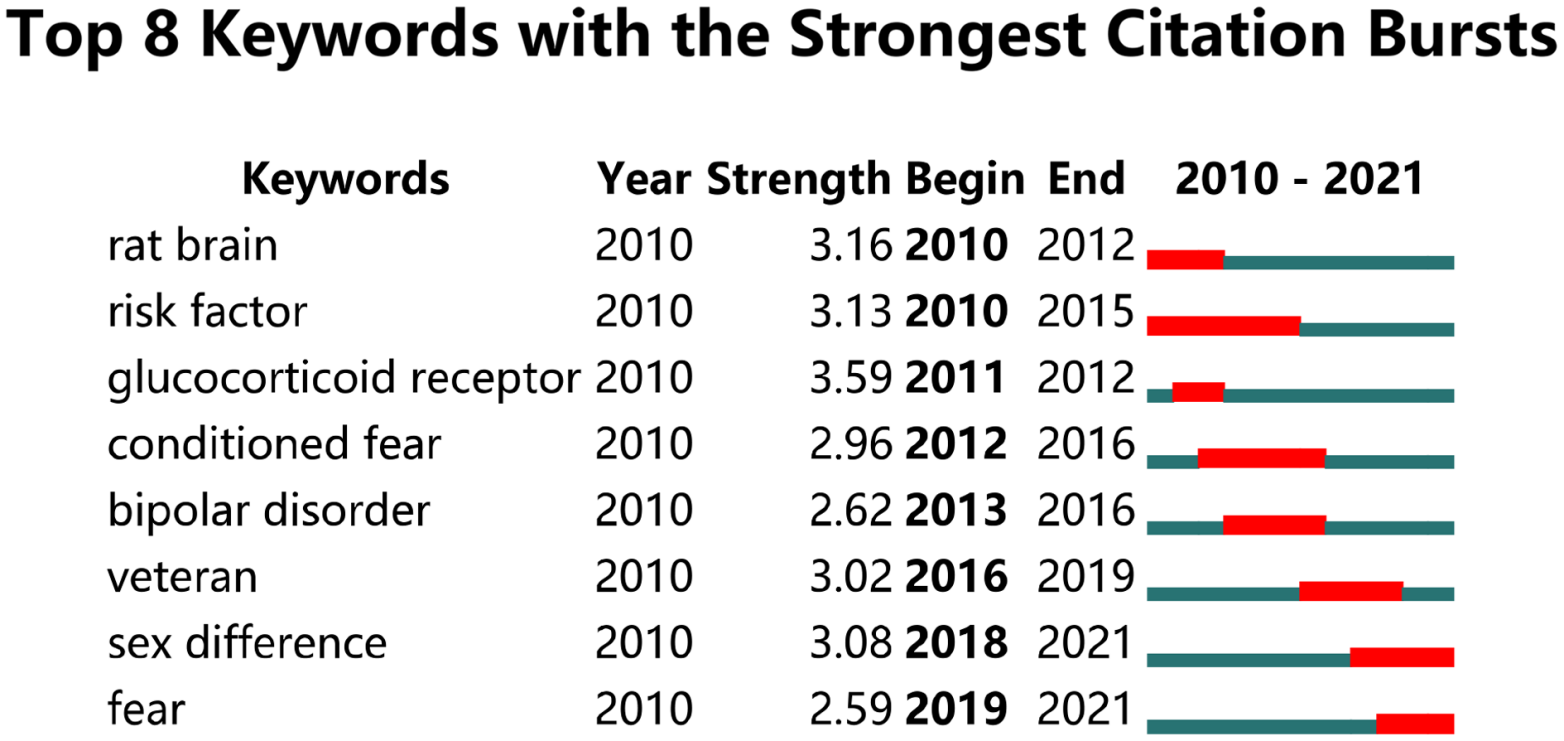
The 8 keywords with the strongest citation bursts in biomarker-related literature.

To explore the study hotspots of biomarker screening in current PTSD research, we clustered the keywords of 418 biomarker-related publications. We identified seven categories, the largest of which was memory, followed by neurodegeneration, PTSD, and glucocorticoid receptor (Figure 9). We also performed burst word extraction on the literature keywords extracted from the biomarker analysis, and extracted eight burst words with a burst strength ranging from 2.59 to 3.59 (Figure 10). “Sex difference” and “fear” were the topics most likely to influence future research on PTSD in forensic medicine; “sex difference” first appeared in 2018 and “fear” first appeared in 2019.

### COVID-19

**Figure 11:**
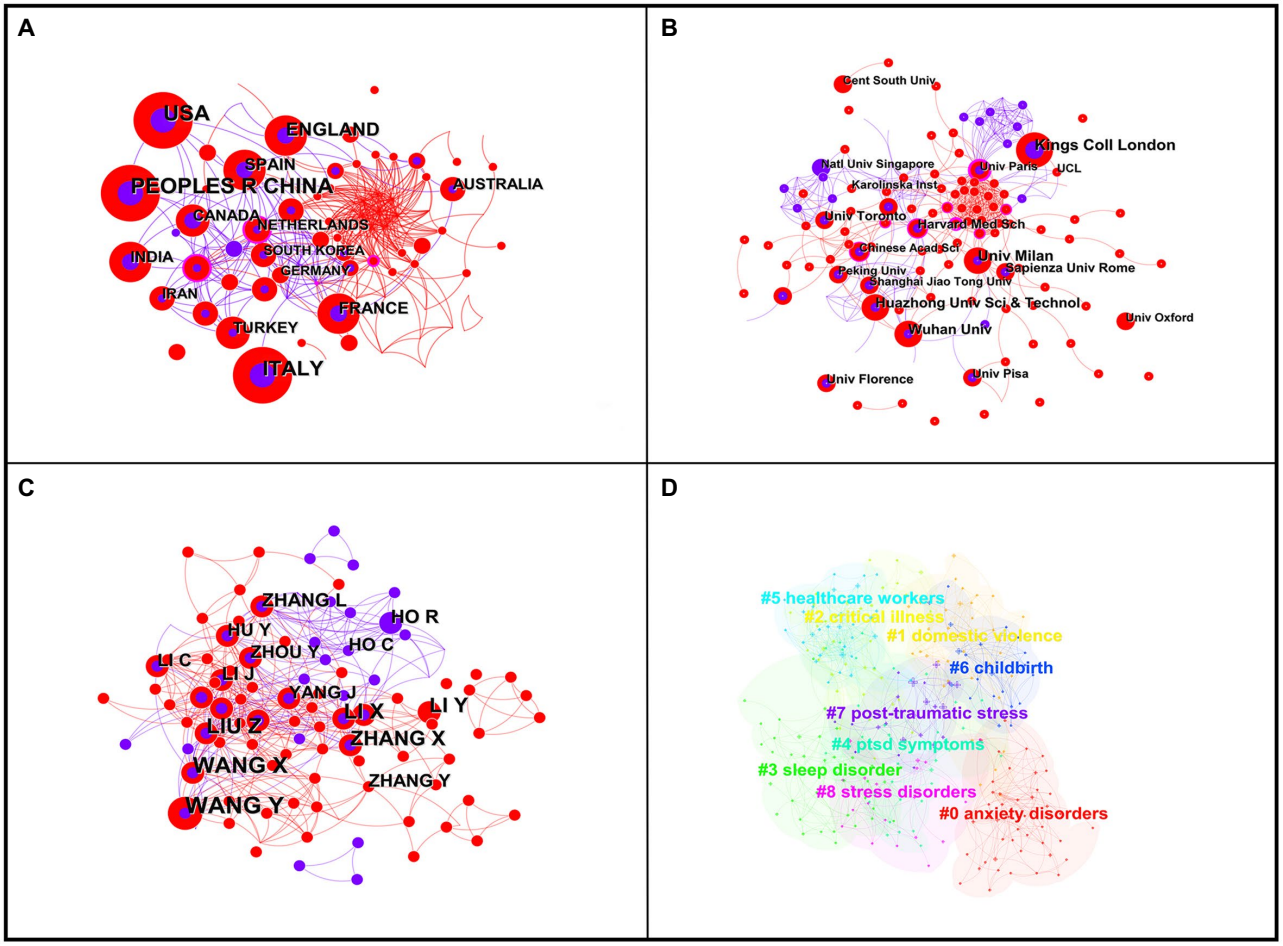
The bibliometric analysis of COVID-19. **(A)** primary countries or regions; **(B)** primary institutions; **(C)** prolific authors; **(D)** keyword clustering.

The serious and prolonged COVID-19 pandemic affected many people physically and/or psychologically. To explore research on the effect of COVID-19 on human stress states, we analyzed COVID-19 research status and hotspots in PTSD-related literature. After screening, 643 relevant articles were identified (169 in 2020 and 474 in 2021). Using CiteSpace to analyze countries or regions, we found that China published the most papers, with 131, followed by the United States with 118 and Italy with 105. Overall, 23 countries had a publication volume ≥ 10, 8 countries had a publication volume ≥ 30, and 4 countries had a publication volume ≥ 50 (Figure 11A). The institution with the most publications was King’s College London, with 21 articles, followed by the University of Milan, with 17 articles. Huazhong University of Science and Technology and Wuhan University were third place with 15 articles each. The top 10 institutions by number of publications were in Italy (Ben et al., 2019), China (Boyd et al., 2018), the United Kingdom, Canada, the United States, Rome, and France (1 each) (Figure 11B). Except for R. Ho, the top 10 authors were all Chinese. Among them, Y. Wang published the most articles related to COVID-19, with 15, followed by X. Wang, with 13 (Figure 11C).

The analysis of top keywords identified 297 nodes and 1,194 links, with a density of 0.0272 (Table 8). The word “mental health” appeared most frequently, with 221 occurrences, ranking first; closely followed by “depression,” which appeared 143 times. As shown in Table 8, five keywords were related to psychological state and two words were related to the participants of COVID-19 studies, namely survivors and healthcare workers. Keyword clustering analysis of the literature related to COVID-19 identified nine categories, of which the highest clustering intensity was for “anxiety disorders,” with “domestic violence” second, and “critical illness” third (Figure 11D). Unfortunately, CiteSpace analysis failed to extract burst words from 297 keywords.

**Table 8:** Top 20 keywords on COVID-19.

Keywords	Articles	Centrality	Year
Mental health	221	0.04	2020
Depression	143	0.01	2020
Impact	124	0.01	2020
Post-traumatic stress disorder	120	0.01	2020
Anxiety	107	0.04	2020
Posttraumatic stress disorder	89	0.05	2020
Psychological impact	87	0.03	2020
Disorder	83	0.01	2020
Stress	71	0.02	2020
Outbreak	71	0.02	2020
Prevalence	67	0.05	2020
Symptom	63	0.05	2020
Survivor	54	0.02	2020
SAR	52	0.01	2020
PTSD	46	0.01	2020
Validation	45	0.03	2020
Psychological distress	44	0	2020
Healthcare worker	42	0.01	2020
Care	42	0.02	2020
Acute respiratory syndrome	41	0.01	2020

## Discussion

### Countries, institutions, journals, authors, co-cited literature, and keywords

The number of PTSD-related publications increased yearly from 2010 to 2021. The growth rate accelerated substantially after 2018, indicating that this research field has received increasing attention by international scholars and has gradually developed into a mature field. Our results show that research in this field was mainly concentrated in developed countries such as the United States and England. The steady development in China continues to improve living standards, and greater attention is being paid to the right to life and health, which explains the increase in research in this field by Chinese scholars. However, more research in this field is needed from other developing countries.

The institution with the most publications was King’s College London, which ranks 33rd in the U.S. News and World Report of Best Global Universities Rankings (U. S. News World Report, 2022) and 37th in the QS World University Rankings (QS World University Rankings, 2022) in 2021–2022. King’s College has the oldest nursing school in the world, its dental school ranks first in the world, and its nursing, psychiatry, and psychology schools rank second in the world. Boston University, Harvard Medical School, Columbia University, and the University of Washington are also world-class universities or research institutions and all provide a good study platform and conditions for PTSD-related research. Among the top 10 institutions, 8 institutions are in the United States, indicating a strong PTSD research output in that country. Although there were many publications from China, these were from a large number of research institutions and related fields. These institutions are widely distributed, and there are as yet no professional and representative research institutions.

The publications reviewed in this study were from 2,821 different journals. Although this is a large number, the distribution was relatively scattered across research areas. The two journals most published in were the comprehensive journals Military Medicine and PLOS ONE, which contained 3.79% of the total published articles. The top 10 journals in terms of publication volume accounted for 14.01% of the total publication volume, with impact factors ranging from 1.470 to 8.396, and there was only one journal in the JCR division Q1. The topics of the top 10 journals were psychology, psychiatry, military medicine, and some comprehensive journals; there were no high quality core professional journals. This may be because the etiology of PTSD is unknown, the symptoms and research participants are diverse, and the scope of the field is relatively wide.

The most published author, S. Lee, is an expert in this field and has conducted in-depth research on PTSD from the aspects of research participants (McLaughlin et al., 2017; Scott et al., 2018), risk factors (Kessler et al., 2014; Bromet et al., 2017), pathogenesis (Kim et al., 2020), diagnosis and treatment (Baek et al., 2019), and epidemiology (McGrath et al., 2017). Research on PTSD by N. Greenberg, N. Fear, and S. Wessely from King’s College London focuses on the mental health of people in environments of war, disaster, and high stress, as well as on diagnosis and treatment (Fear et al., 2010; Greenberg et al., 2015; Brooks et al., 2020; Irizar et al., 2021). Among the highly cited authors, an article by R.C. Kessler, “Twelve-month and lifetime prevalence and lifetime morbid risk of anxiety and mood disorders in the United States” (Kessler et al., 2012), had 1,358 citations. The article “Mental disorders, comorbidity and suicidal behavior: Results from the National Comorbidity Survey Replication” (Nock et al., 2010) has also been cited 687 times. The single most cited article of E.B. Foa was “Cognitive-behavioral therapy for anxiety disorders: An update on the empirical evidence” (Kaczkurkin and Foa, 2015), which received 124 citations.

This study extracted nine turning points from the literature, suggesting that neural network centers, the HPA axis, and biomarkers are new study directions. The investigation of turning points analyzes the co-citation of documents in a specific field. The extraction of turning points can help to identify future developmental directions of a field and the articles that have had the greatest influence (Li et al., 2022). Turning points usually connect two or more clusters, indicating key nodes for new study; higher centrality nodes represent emerging new research hotspots (Li et al., 2022). Of the nine turning point articles, six discussed the role of neural network hubs and the HPA axis in PTSD research (Milad et al., 2009; Brewin et al., 2010; Shin and Liberzon, 2010; Pitman et al., 2012; van Zuiden et al., 2012; Daskalakis et al., 2013) and one article explored the role of the pituitary adenylyl cyclase activating polypeptide PAC1 receptor pathway in human stress responses (Ressler et al., 2011). Thus, eight of the nine turning point articles were on neural network centers, the HPA axis, and biomarkers, indicating that the search for iconic SRAs and molecular diagnostic markers has been a research direction over the past 12 years.

Analysis of the co-occurrence of keywords, excluding the search term “PTSD,” showed that the top five keywords were “symptom,” “depression,” “mental health,” “prevalence,” and “disorder,” which represent the hotspots that have received the most attention in the current study field. Keyword cluster analysis showed that “amygdala,” “intimate partner violence,” and “Iraq” were the most popular PTSD topics. In addition, analysis of the top 25 burst words showed that “rat model,” “mental health,” and “satisfaction” were the keywords most likely to have a clear impact on future research in this field. Except for “amygdala,” other SRAs did not feature in these keywords, which may be because they relate to basic experimental topics in PTSD and do not appear in the keywords of the publications, or because they involve multiple SRAs, and so were replaced by words such as “central nervous system” or “brain.”

### Research hotspots

#### SRA

In earlier PTSD studies, research on SRAs focused on the hippocampus, which plays a role in learning, memory, spatial navigation, emotional processing, and facilitating the neuroendocrine coping response to stress. Increased glucocorticoid concentrations in the brain after stress have been shown to damage hippocampal neurons in animal models (Zhang et al., 2012; Kim et al., 2015), and atrophy of hippocampal tissue volume has been found in PTSD patients (Henigsberg et al., 2019). The focus on SRAs has gradually expanded to circuits related to the prefrontal cortex, amygdala, insula, and cingulate gyrus (Pitman et al., 2012; Yehuda et al., 2015). Some studies have shown that both trauma-and non-trauma-related stress can reduce activation of the anterior cingulate gyrus and prefrontal cortex in PTSD patients; similarly, trauma-and non-trauma-related emotional stimuli can induce amygdala activation in PTSD patients (Hayes et al., 2012; Pitman et al., 2012; Sartory et al., 2013). In addition, one study showed that the blood oxygen level dependent (BOLD) signal intensity of the amygdala and hippocampus increased and the BOLD signal intensity of the dorsal prefrontal cortex decreased during a facial expression task in PTSD patients compared with healthy individuals (Lee et al., 2021). Following evocation of an emotional response, the BOLD signal intensity in the left anterior insula, anterior cingulate gyrus, and bilateral frontal regions decreased, whereas the BOLD signal intensity in the left parahippocampal region increased in PTSD patients (Lee et al., 2021).

We found that the two SRAs with the most publications involved the amygdala (48.46%) and the prefrontal cortex (48.17%). The amygdala plays an important role in detecting threats, learning fears, expressing fears, and enhancing memory for emotional events (Pitman et al., 2012; Henigsberg et al., 2019). Functional neuroimaging has shown that amygdala activation is exaggerated when participants are exposed to trauma-related or general stimuli, and PTSD patients show greater amygdala activation than controls if they have acquired a conditioned fear response (Pitman et al., 2012). As an advanced regulatory center, the prefrontal cortex also shows a major response to stress. A voxel-based morphometry analysis showed that patients with chronic PTSD had gray matter structural damage in the prefrontal cortex, occipital cortex, and parietal cortex; after symptoms of the disorder improved, gray matter structural damage was alleviated but did not return to the level of trauma control (Tan et al., 2013). The present findings indicate that many publications in the field have focused on the hippocampus, and the growth rate of such publications is the highest in recent years, indicating that the hippocampus plays an important role in the pathophysiology of PTSD (Hughes and Shin, 2011; Yehuda et al., 2015; Henigsberg et al., 2019). Studies have shown that neural network research involving multiple SRAs simultaneously is more conducive to an in-depth and dynamic understanding of PTSD, and may become an important research hotspot in the future (Shin and Liberzon, 2010; Yehuda et al., 2015).

Analysis of SRA-related literature showed that three of the six keywords with a frequency of ≥200 were SRAs (“prefrontal cortex,” “medial prefrontal cortex,” “amygdala”), which further indicates that the amygdala and prefrontal cortex play an important role in PTSD research. Keyword cluster analysis showed that current key research topics in the SRA research field include “functional connectivity,” “elimination,” “single prolonged stress,” “panic disorder,” “risk,” and “individual differences.” Additionally, “rat model,” “oxidative stress,” “gender difference,” “reconsolidation,” “deficit,” “transcranial magnetic stimulation,” “risk,” and “acid amide hydrolase” were the topics most likely to influence future research on PTSD in forensic medicine. This shows that the research direction of SRAs in PTSD is becoming more extensive, which will help to generate a more thorough understanding of PTSD. Recently, a more comprehensive PTSD neural circuit model has been proposed that comprises four parts: frontal limbic circuit, default mode network, salience network, and central executive network (Sripada et al., 2012; Yehuda et al., 2015). This neural circuit can better explain the different stress states of PTSD patients that are caused by different stress factors (Daniels et al., 2010; Rabellino et al., 2015). These studies show that different SRAs are involved in different PTSD phenotypes, with different brain circuit activity and connectivity patterns, and indicate that researchers should consider the problem of heterogeneity when studying this disease.

### Biomarkers

It is important to identify pre-traumatic and post-traumatic-related biological changes in PTSD and to determine the role of these markers in PTSD. Our findings showed that research on PTSD biomarkers has been increasing in recent years. However, only two of the top 10 keywords were identified as biomarkers (“DNA methylation” and “glucocorticoid receptor”). For DNA methylation, A.K. Smith et al. found that global methylation was increased and CpG sites were differentially methylated in five genes (TPR, CLEC9A, APC5, ANXA2, and TLR8) in PTSD patients. A CpG site in the NPFFR2 gene was associated with total life stress, and plasma levels of interleukin (IL)-4, IL-2, and tumor necrosis factor-alpha were also associated with PTSD, child abuse, and total life stress, suggesting that psychosocial stress may be associated with peripheral immune dysregulation global and gene-specific DNA methylation patterns (Smith et al., 2011). The glucocorticoid receptor is a transcription factor that binds to the glucocorticoid response element and activates its transcription. Glucocorticoid is the main factor that affects the stress response; abnormal glucocorticoid signals are associated with an increased risk of psychological and mood disorders, including schizophrenia, PTSD, and depression (Yehuda et al., 2015; Mourtzi et al., 2021). The keyword clustering results also showed that the glucocorticoid receptor was a hot biomarker in current PTSD research. Of the eight burst words, “gender difference” was the topic most likely to have an impact on future research in this field. Studies have shown sex-related changes in gene expression in PTSD patients, such as alterations in the serotonergic (Goswami et al., 2010), somatostatin (Tripp et al., 2011), and galanin systems (Barde et al., 2016). Within the neuropeptide system, the corticotropin-releasing factor system selectively alters corticotropin-releasing factor binding protein mRNA levels in the amygdala of depressed patients and in bipolar male, but not female, patients (Brivio et al., 2020).

Our review and analysis of the relevant literature on biomarkers showed that PTSD biomarkers are mainly divided into the following categories: (i) HPA axis and sympathetic nervous system hormone secretion: when the body is in a state of stress, there may be an abnormal release of glucocorticoids and catecholamines and a unique set of HPA axis changes that are sensitive to negative feedback regulation in PTSD (Bhattacharya et al., 2019). Currently, glucocorticoids and glucocorticoid receptors have been postulated as important factors in the etiology and pathophysiology of PTSD. (ii) Immune and inflammatory responses: during stress injury, the number of peripheral blood immune cells (CD4^+^ T cells, CD8^+^ T cells) is increased in PTSD patients, and the secretion of inflammatory cytokines (IL-2, IL-6, IL-8, tumor necrosis factor) in serum is abnormal (Bonne et al., 2011; O'Donovan et al., 2013). (iii) Endocannabinoids and neurosteroids: there are substantial changes in anandamide and 2-arachidonyl glycerol in the blood of PTSD patients (Berardi et al., 2016; Kirchhoff et al., 2019); neurosteroids such as allopregnanolone have marked inhibitory effects on glucocorticoid and norepinephrine signaling (Tomaselli and Vallee, 2019). (iv) Neurotransmitter secretion: PTSD is associated with imbalances in endogenous neuronal substance secretion, such as brain-derived neurotrophic factor, gamma-aminobutyric acid, neuropeptide Y, and glutamate (Zhang G. Q. et al., 2014; Ke et al., 2020). (v) PTSD-related genes: genetic activity related to the HPA axis, the noradrenergic system, and the limbic amygdala frontal pathway, such as FKBP5 polymorphism and demethylation, has been found to mediate fear processing in PTSD-related gene studies (Kang et al., 2019; Yun et al., 2022). Although current research on biomarkers in the field of PTSD has achieved some results, the present analysis of relevant literature shows that research on biomarkers in this field has just begun. These biomarkers are considered potential therapeutic targets but findings need to be replicated using more robust studies and confirmed using large-scale, multiomic, and genome-wide epigenetic studies.

### COVID-19

The outbreak of COVID-19 in 2019 substantially affected the global economy and daily life. Although the pandemic was gradually controlled, the psychological distress it caused continues, and severe cases of such distress can lead to PTSD (Dubey et al., 2020; Liang L. et al., 2020; Liang X. et al., 2020). The number of relevant publications screened in this study was 643 for the 2-year period 2020–2021, which indicates that scholars are very concerned about the status of mental health and PTSD during the COVID-19 pandemic. The countries with the largest number of publications were China (131) and the United States (118), which may be because these countries saw the earliest outbreaks of COVID-19 and a higher number of infections. Except for R. Ho, the top 10 authors were all Chinese, which reflects a higher scientific research capability in China and a greater emphasis on people’s physical and mental health.

Of the top 20 keywords, 5 were related to psychological states. Studies have shown that in China, approximately 12.8% of adolescents have PTSD symptoms and psychological distress after the COVID-19 pandemic (Liang L. et al., 2020; Liang X. et al., 2020). A highly cited article in the field reported relatively high rates of symptoms of anxiety (6.33 to 50.9%), depression (14.6 to 48.3%), PTSD (7 to 53.8%), psychological distress (34.43 to 38%), and stress (8.1 to 81.9%) in the general population during the COVID-19 pandemic in China, Spain, Italy, Iran, the United States, Turkey, Nepal, and Denmark (Xiong et al., 2020). In the present study, the collinearity of keywords showed that the research participants of COVID-19 studies were mainly survivors (Chamberlain et al., 2021) and healthcare workers (Chew et al., 2020). To comprehensively explore the mental health status of different groups of people during the pandemic, surveys have been conducted on older people (Srifuengfung et al., 2021), adolescents (Liang L. et al., 2020; Liang X. et al., 2020), pregnant women (Kara et al., 2021), family members of patients (Carson et al., 2021), and other groups.

We found that studies on PTSD associated with the COVID-19 pandemic showed the following main characteristics: (i) Population studies mostly used self-administered questionnaires for data collection, which can make the data less representative (Wang et al., 2020). (ii) Most of the studies focused on special groups, such as patients (Craparo et al., 2022), medical staff (Batra et al., 2020), or students (Li et al., 2021), and there were fewer studies on the larger general population. (iii) Most of the findings were regional (Zhang and Ma, 2020), and the worldwide prevalence of PTSD caused by COVID-19 remains unknown. (iv) Most of the studies on PTSD caused by the COVID-19 pandemic have been epidemiological investigations focusing on mental health; the negative effects of stress on the body, the mechanism of damage, and the relationship between COVID-19 and the stress response require further research. However, it is undeniable that COVID-19 not only has direct serious pathological consequences but may also cause psychological stress and induce severe mental illness, especially PTSD.

### Forensic medicine

Due to the particularity of forensic medicine, forensic practitioners pay more attention to the pathophysiological changes of PTSD, injury sites, forensic diagnosis, and the relationship between PTSD and injury (disease, death) (Byard, 2021), which is why this study focuses on the molecular markers and neural circuits of PTSD. In litigation cases involving PTSD, the reliability of the obstacle assessment conclusion depends more or less on the recognition and identification ability of judicial experts, insurance company employees, and defense lawyers. Therefore, not only clinicians but also all relevant experts should strive to master their diagnostic criteria, understand the knowledge of identifying camouflage PTSD, enrich the practical experience and minimize miscarriage of justice (Kulich et al., 2000; Kleinman and Martell, 2015). However, the statistical analysis of PTSD-related literature found that most of them came from journals in the fields of psychiatry, psychology, and neuroscience, while there were few journals in the field of forensic medicine, indicating that PTSD mainly focused on clinical and basic research, and the research on PTSD in forensic medicine needs to be further strengthened. We believe that the key PTSD research foci in the field of forensic medicine are as follows: the acquisition of a more systematic understanding of the abnormal activation of neural circuits in PTSD patients, the personalization of forensic diagnosis, the evaluation of pre-traumatic risk factors, the intensity of different stressors, the degree of the stress response, the long-term repeated trauma experience, the relationship of PTSD to injury and disease, and individual differences.

## Conclusion

The present results show that research on PTSD in forensic medicine has been steadily increasing, mostly in developed countries. Of the 2,821 journals involved in this research, 13 were forensic-related journals; however, the distribution of journals is relatively scattered and there is a lack of professional core journals. Neural network hubs, the HPA axis, and biomarkers are research hotspots and most likely to have a clear impact on future research. COVID-19 can cause severe psychological stress and induce PTSD, but this relationship requires further study. The literature on SRAs and biomarkers has gradually increased over time, but owing to the influence of various factors, the systemic neural brain circuits and specific biomarkers of PTSD have not been identified. Currently, attempts are being made to screen PTSD risk-related biomarkers and molecular targets using multiomics and molecular biology techniques, which are very useful to explore the pathophysiological changes in PTSD and could help to establish PTSD-related molecular networks. Moreover, given the complexity of human genetics, cultural diversity, the social environment, and other risk factors, methods must be found to obtain more diverse samples of patients from different backgrounds.

## Data availability statement

The original contributions presented in the study are included in the article/supplementary material, further inquiries can be directed to the corresponding authors.

## Author contributions

WZ and YL completed data statistical analysis and manuscript writing. XM, HY, ZW, and RS searched and organized the database. BC and WS supervised the design and revised the manuscript. All the authors read and approved the final version of the manuscript.

## Funding

This study was supported by the Key National Natural Science Foundation of China (82130055), National Natural Science Foundation of China (82072109 and 81971787), and the Key Research and Development Projects in Hebei Province (20377722D).

## Conflict of interest

The authors declare that the research was conducted in the absence of any commercial or financial relationships that could be construed as a potential conflict of interest.

## Publisher’s note

All claims expressed in this article are solely those of the authors and do not necessarily represent those of their affiliated organizations, or those of the publisher, the editors and the reviewers. Any product that may be evaluated in this article, or claim that may be made by its manufacturer, is not guaranteed or endorsed by the publisher.

## Footnotes

^1^ https://www.webofscience.com/wos/woscc/basic-search

^2^ https://sourceforge.net/projects/citespace/files/latest/download
